# Supplementary material for: Hierarchical Bayesian myocardial perfusion quantification
Source: Med Image Anal. Author manuscript; Available in PMC 2021 Jul 12. (PMC6880627; doi:10.1016/j.media.2019.101611)
Supplement: Appendix [file EMS128751-supplement-Appendix.pdf]

Appendix

A.1. Residue function

The pair of coupled differential Eqs. (1) and (2) can be solved analytically using the Laplace transform to yield a solution in the form:

$$C_{\Theta}(t) = R_F(t, \Theta) * C_{AIF}(t - \tau_0)$$

The residue function R_F is given as: $R_F(t, \Theta) = A\ \exp(\alpha t) + (1 - A)\ \exp(\beta t)$, where:

$$\alpha, \beta = \frac{1}{2}\left[-\left(\frac{PS}{v_p} + \frac{PS}{v_e} + \frac{F_p}{v_p}\right) \pm \sqrt{\left(\frac{PS}{v_p} + \frac{PS}{v_e} + \frac{F_p}{v_p}\right)^2 - 4\frac{PS}{v_e}\frac{F_p}{v_p}}\right]$$

$$A = \frac{\alpha + \frac{PS}{v_p} + \frac{PS}{v_e}}{\alpha - \beta}$$
